# Novel *WEE2* compound heterozygous mutations identified in patients with fertilization failure or poor fertilization

**DOI:** 10.1007/s10815-021-02285-z

**Published:** 2021-09-03

**Authors:** Jiamin Jin, Xiaomei Tong, Yin-Li Zhang, Weijie Yang, Yerong Ma, Peipei Ren, Feng Zhou, Songying Zhang

**Affiliations:** 1 Assisted Reproduction Unit, Department of Obstetrics and Gynecology, Sir Run Run Shaw Hospital, Zhejiang University School of Medicine, No. 3 Qingchun East Road, Jianggan District, Hangzhou, 310016 China; 2 Key Laboratory of Reproductive Dysfunction Management of Zhejiang Province, Hangzhou, 310016 China

**Keywords:** WEE2, Fertilization failure, Pronucleus formation, Novel mutation

## Abstract

**Purpose:**

To study associations between novel *WEE2* mutations and patients with fertilization failure or poor fertilization.

**Methods:**

Thirty-one Chinese patients who underwent treatment with assisted reproductive technology and suffered from repeated (at least two times) total fertilization failure (TFF) or a low fertilization rate were enrolled. Genomic DNA was extracted from patients for whole-exome sequencing. Suspicious mutations were validated by Sanger sequencing. WEE2 protein levels in oocytes from affected patients were examined by immunofluorescence. Disruptive effects of mutations on WEE2 protein stability, subcellular localization, and kinase function were analyzed through western blotting, immunofluorescence, and flow cytometry in HeLa cells.

**Results:**

Three of thirty-one (9.6%) enrolled patients had six compound heterozygous mutations of the *WEE2* gene, and three of them were reported here for the first time (c.115_116insT, c.756_758delTGA, and c.C1459T). Oocytes from affected patients showed decreased WEE2 immunofluorescence signals. In vitro experiments showed that the mutant *WEE2* gene caused reduced WEE2 protein levels or cellular compartment translocation in HeLa cells, leading to decreased levels of the phosphorylated Cdc2 protein. Compared with the wild-type WEE2 protein, the mutant WEE2 proteins were also found to have different effects on the cell cycle.

**Conclusion:**

Three novel compound heterozygous *WEE2* variants were found in patients with pronucleus formation failure. This study provides new evidence that *WEE2* mutations result in loss of function, which could result in fertilization failure.

**Supplementary Information:**

The online version contains supplementary material available at 10.1007/s10815-021-02285-z.

## Introduction

Fertilization is a complex process that involves multiple steps and is very important for mammalian reproduction. For mammalian fertilization, meiosis II (MII) oocytes are activated by spermatozoa, which induce a series of oscillations in cytosolic-free Ca2 + [1]. Calmodulin-dependent protein kinase II is activated by Ca2 + oscillations and phosphorylates WEE2 to inactivate maturation promoting factor (MPF) [2]. The fertilization process is finally accomplished by cortical granule exocytosis, pronucleus (PN) formation, and extrusion of the second polar body [3].

In the treatment of in vitro fertilization (IVF), 5–15% of couples with normospermia have total fertilization failure (TFF), and 20% have low fertilization rates (defined as < 25%) [4]. Penetration problems of the zona pellucida (ZP) are thought to be the major reason for fertilization failure in IVF, and these problems can be treated by intracytoplasmic sperm injection (ICSI). However, 1–5% of couples still suffer from TFF after ICSI [5]. In addition to iatrogenic defects, some pathogenic genetic mutations have been found in these patients [6]. In 2018, four patients from consanguineous families were found to have homozygous mutations in *WEE2*, and their oocytes presented no PN formation but had normal extrusion of the second polar body after ICSI [7]. This study was the first to show the potential associations between *WEE2* mutations and human TFF.

In mice, mWee2, also known as mWee1B, is a homolog 2 of mWee1 and is highly expressed in the ovaries [8]. This kinase participates in maintaining arrest of oocyte meiosis and the fertilization process by controlling stabilization/destabilization of MPF [8]. MPF comprises cyclin B and Cdc2-kinase. In arrest of oocyte meiosis, mWee2 is phosphorylated in the presence of high cyclic AMP levels, leading to phosphorylation of Cdc2-kinase, which inhibits activation of Cdc2-kinase. Inactivated Cdc2 maintains inactivation of MPF, and mouse oocytes remain in the germinal vesicle (GV) state. After the luteinizing hormone (LH) surge, mWee2 phosphorylation is disrupted by decreased cyclic AMP levels, which increases MPF kinase activity and initializes re-entry of oocytes into the meiotic cell cycle [8]. mWee2 is reactivated by Ca^2+^ oscillations when oocytes are penetrated by sperm. After reactivation of mWee2, Cdc2 is phosphorylated, and MPF is inactivated again. Finally, oocytes exit from MII, and the PN is formed [2, 9]. In recent years, *WEE2* gene mutations have been discovered in patients with TFF and low fertilization rates [10–15]. However, the specific mechanism of WEE2 in human fertilization remains unclear, and novel mutations need to be identified.

In the present study, we enrolled typical patients who had repeated TFF or a low fertilization rate in IVF and ICSI treatments. These patients underwent whole-exon sequencing, and novel *WEE2* variants were found. These variants may contribute to failed PN formation and fertilization failure in these infertile individuals.

## Material and methods

### Patients

Thirty-one patients were recruited because of TFF or low fertilization rates during assisted reproductive technology (ART) treatment at Sir Run Run Shaw Hospital. The inclusion criteria were as follows: (1) primary infertility for longer than 2 years; (2) at least two ART treatments, including IVF and ICSI, ending with TFF or low fertilization (< 25% MII); (3) normal male sperm examinations; and (4) normal karyotype for the couple. Peripheral blood was extracted from patients for whole genome DNA. To compare WEE2 protein levels in different patients, MII oocytes derived from in vitro maturation (IVM) were donated by healthy patients, and 0PN embryos were acquired from affected patients. All human samples were collected after informed consent was obtained.

### Genetic studies

Whole-exon analysis was performed on peripheral blood samples. Whole-exome capture (Agilent, Santa Clara, CA, USA) and sequencing analysis (Illumina, San Diego, CA, USA) were conducted by iGeneTech Company (Beijing, China). Briefly, 200 ng of genomic DNA from each individual was sheared by a Bioruptor (Diagenode, Liege, Belgium) to acquire 150–200-bp fragments. The ends of the DNA fragment were repaired, and Illumina Adaptor was added (Fast Library Prep Kit; iGeneTech). After a sequencing library was constructed, whole exons were captured with the AIExome Enrichment Kit V1 (iGeneTech) and sequenced on an Illumina platform (Illumina) with 150-base paired-end reads. Raw reads were filtered to remove low-quality reads by using FastQC. Clean reads were then mapped to the reference genome GRCh37 by using Bwa. After removing duplications, SNVs and InDels were called and annotated by using GATK. All suspicious mutations met the following inclusion criteria: (1) mutation frequencies < 0.1% in public databases, including the East Asian population of the 1000 Genome and Exome Aggregation Consortium (ExAC) databases; (2) homozygous and compound heterozygous recessive mutations or heterozygous dominant mutations; (3) mutations were exonic nonsynonymous, splice site variants or coding indels; and (4) mutated genes reported as important in fertilization in human and/or animal models. Whole-exome sequencing data from the three affected patients are shown in Table S1.

Suspicious mutations were validated by Sanger sequencing. Briefly, DNA was obtained from peripheral blood using a standard protocol (MiniBEST Universal Genomic DNA Extraction Kit, 9756; TaKaRa, Kusatsu, Japan). Sanger analysis was conducted with KAPA HiFi HotStart ReadyMix (kk2602; Kapa Biosystems, Boston, MA, USA) and the ABI 3100 DNA analyzer (Applied Biosystems, Foster City, CA, USA). The primers used for Sanger analysis are shown in Table S2.

### Expression plasmid construction and mutagenesis

Immature oocytes donated by healthy women were used to extract the full-length *WEE2-*coding DNA. The pDONR201 vector was used to construct an entry clone, and the pDRF-FLAG vector was used to construct an expression clone under the instructions of Gateway Technology (12,535–019; Invitrogen, Carlsbad, CA, USA). Specific site-directed mutagenesis of the pDONR201-WEE2 entry clone was conducted using the protocol from the QuikChange Multi Site-Directed Mutagenesis kit (200,513; Agilent). The primers used in mutagenesis are shown in Table S3.

### Immunofluorescence

Unfertilized oocytes from affected individuals and IVM oocytes that were donated by healthy patients were fixed with 2% paraformaldehyde. HeLa cells transfected with plasmids were fixed with 4% paraformaldehyde. Oocytes were incubated with WEE2 antibody (ab121943, dilution: 1:100; Abcam, Cambridge, UK) and HeLa cells were incubated with FLAG antibody (8146, dilution: 1:1000; CST, Beverly, MA, USA) overnight at 4 °C. Oocytes and HeLa cells were counterstained with fluorescent dye-conjugated secondary antibodies (dilution: 1:200, A11036 for oocytes; dilution: 1:1000, A11034 for HeLa cells; Thermo Scientific, Waltham, MA, USA) and Hoechst 33,342 (dilution: 1:1000, H3570; Invitrogen). Digital images were acquired with a fluorescence confocal microscope (LSM800; Carl Zeiss, Jena, Germany).

### Western blot analysis and compartment fractionation

Mutant and wild-type *WEE2* plasmids were transfected into HeLa cells with Lipofectamine 3000 Reagent according to the manufacturer’s instructions (L3000008; Invitrogen). After 36 h of incubation, transfected cells were lysed and subjected to SDS-PAGE. Primary antibodies against FLAG (8146, dilution: 1:1000; CST) and pY15-Cdc2 (4539, dilution: 1:1000; CST) were used to detect the mutation effects of pDRF-FLAG-WEE2 plasmids. Primary antibodies against GAPDH (60,004, dilution: 1:20,000; Proteintech, Wuhan, China) and Cdc2 (77,055, dilution: 1:1000; CST) were used as internal controls. The secondary antibodies, horseradish peroxidase-linked anti-rabbit and anti-mouse IgG (7074 and 7076, dilution: 1:2000; CST), were incubated with the membranes. Finally, bound proteins were visualized by enhanced chemiluminescence (WBKLS0500; Millipore Corp., Billerica, MA, USA).

To determine the effects of *WEE2* mutant variants on subcellular localization, compartment extraction of transfected HeLa cells was conducted with the Nuclear and Cytoplasmic Extraction Reagents Kit (78,833, Thermo Scientific). The nuclear and cytoplasmic compartments were quantified by western blotting using antibodies against Lamin B1 (66,095–1-lg, dilution: 1:10,000; Proteintech) and GAPDH (60,004, dilution: 1:20,000; Proteintech) to normalize the quantities of nuclear and cytoplasmic proteins, respectively.

### Flow cytometry

The effects of overexpression of wild-type and mutant WEE2 protein on cell cycle distribution were assessed by flow cytometry with propidium iodide (V13245; Thermo Scientific) and RNase I solution (D7073; Beyotime, Shanghai, China). Cell cycle distribution data analysis was performed with the FACSCalibur system (ACS101; BD Biosciences, San Diego, CA, USA) and ModFit LT software (Verity Software House, Topsham, ME, USA).

### Statistical analysis

Western blotting, immunofluorescence, and flow cytometry results are representative of at least three independent experiments. Significance was determined by one-way ANOVA (*p* < 0.05). Quantitation of the intensity of immunofluorescence images and western blot bands was analyzed with ImageJ software (version 1.51j8; Wayne Rasband, National Institutes of Health, Bethesda, MD, USA).

## Results

### Genetic characteristics of *WEE2* mutations

**Fig. 1:**
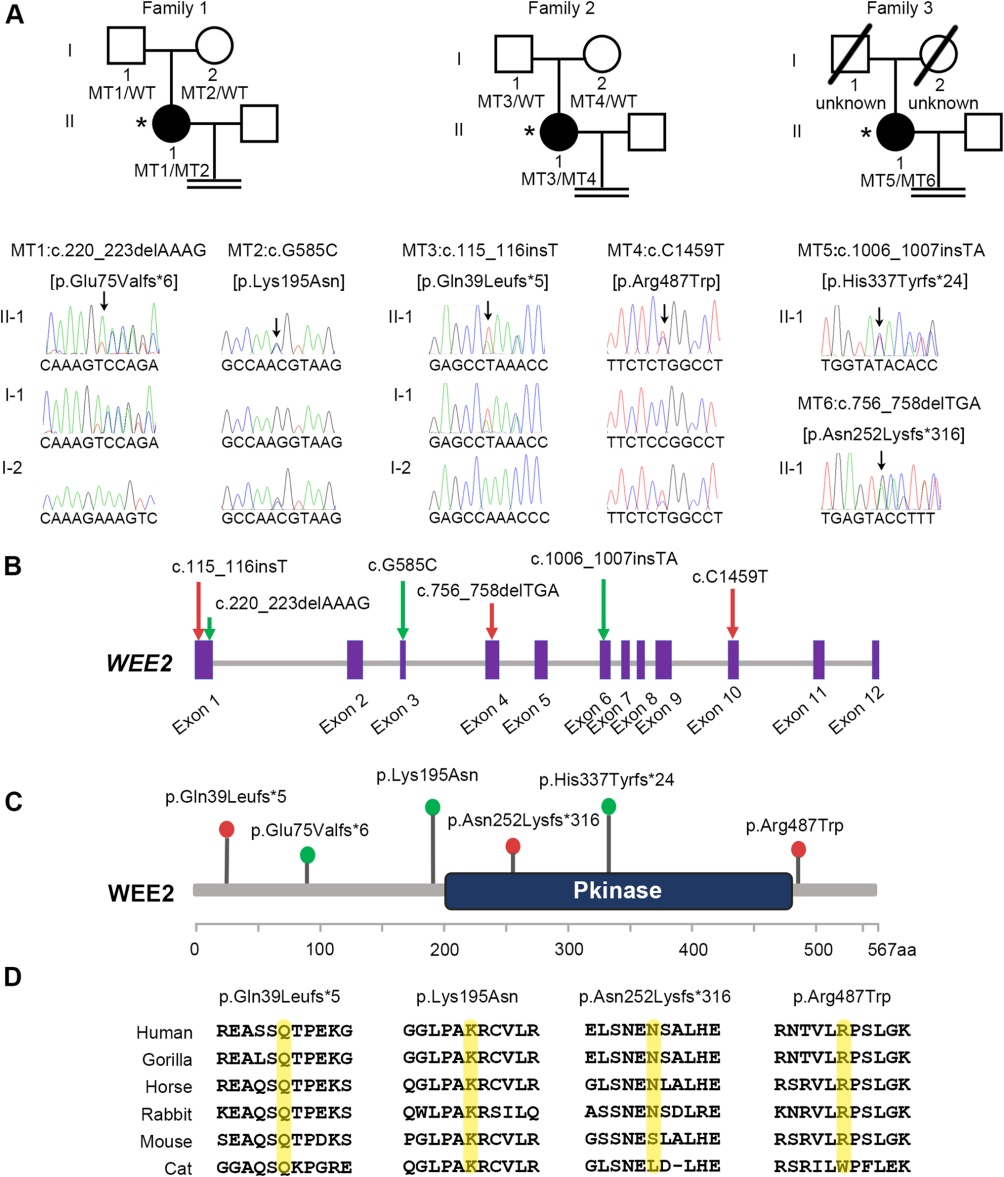
Identification of *WEE2* mutations in three patients and analysis of location and conservation of mutant residues. (**A**) Pedigree analysis of three patients shows total fertilization failure or poor fertilization. Affected individuals are indicated by asterisks and black circles. The “ = ” sign indicates infertility. Sanger sequencing results are shown below the pedigrees. The start of the mutant bases is marked with a black arrow. Sanger analysis of I-1 and I-2 from family 3 was not available. Positions of *WEE2* mutations in the genomic structure (**B**) and protein structure (**C**). Red arrows represent novel mutations discovered in our study, and green arrows indicate mutations that were identified by others but also found in our study. (**D**) Conservation analysis of mutant amino acids, which were compared in six mammalian species

We found that three patients were carriers of compound heterozygous *WEE2* mutations (Fig. 1A). The carrier rate of *WEE2* gene mutations in patients with TFF or low fertilization rates at our center was 9.6% (3/31). Detailed information on whole-exome sequencing is shown in Table S1. The mutational sites were confirmed by Sanger sequencing in each proband patient and the patient’s parents, except for family 3, for which the parents were not available. We found that they all had compound heterozygous mutations, and the mutations from families 1 and 2 had a recessive inheritance pattern.

Half of the six mutation sites were novel (c.115_116insT, c.C1459T, and c.756_758delTGA) and the other half (c.G585C, c.220_223delAAAG, and c.1006_1007insTA) have been reported previously [7, 10, 12]. These mutation sites were labeled on the schematic map of the *WEE2* gene (Fig. 1B). Two mutation sites were located in the kinase domain, and four other mutations were located in other parts of *WEE2* (Fig. 1C). The proband in family 1 had the previously reported c.220_223delAAAG (p.Glu75Valfs*6) mutation and the missense mutation c.G585C (p.Lys195Asn). Patient II had one novel frameshift protein-truncating mutation, c.115_116insT (p.Gln39Leufs*5), and one novel missense mutation, c.C1459T (p.Arg487Trp). Patient III had two compound heterozygous mutations, including one novel c.756_758delTGA (p. Asn252Lysfs*316) mutation and one previously reported c.1006_1007insTA (p. His337Tyrfs*24) mutation. The allele frequency of p.Arg487Trp was 0.0006 in the ExAC database, while the other five mutations were not found in the ExAC database. None of the mutations was found in the 1000 Genome database. Detailed information on the mutations is shown in Table 1. Conservation analysis in different species is also shown in Fig. 1D.

**Table 1:** Overview of the *WEE2* mutations observed in three families

Patients	Genomic position on chr. 7 (bp)	Exon	cDNA change	Protein change	Mutation type	SIFT^a^	PolyPhen2^a^	1000genome^b^	ExAC^b^
1	141,408,778	1	c.220_223delAAAG	p.Glu75Valfs*6	Frameshift	-	-	NA	NA
	141,416,067	3	c.G585C	p.Lys195Asn	Missense	D	P	NA	NA
2	141,408,673	1	c.115_116insT	p.Gln39Leufs*5	Frameshift	-	-	NA	NA
	141,427,170	10	c.C1459T	p.Arg487Trp	Missense	D	B	NA	0.0006
3	141,419,042	4	c.756_758delTGA	p.Asn252Lysfs*316	Frameshift	-	-	NA	NA
	141,423,059	6	c.1006_1007insTA	p.His337Tyrfs*24	Frameshift	-	-	NA	NA

Abbreviations: *cDNA*, complementary DNA

^a^Mutation assessment by SIFT and PolyPhen2. *D*, damaging; *P*, probably damaging; *B*, benign

^b^Frequency of corresponding mutations in the East Asian population of the 1000 Genome and ExAC database

### Clinical characteristics of patients with *WEE2* mutations

Detailed clinical characteristics of the three patients with *WEE2* mutations are shown in Table 2.

**Table 2:** Clinical characteristics of affected individuals

Patients	Age (years)	Infertility duration (years)	IVF/ICSI cycles	Total oocytes	GV oocytes	MI oocytes	MII oocytes	Fertilized oocytes	Normal fertilized oocytes	Cleavage embryos
1	29	7	IVF	14	0	0	14	0	0	0
	29	7	ICSI^a^	14	0	1	11	0	0	0
2	30	3	IVF^b^	19	0	0	19	1	0	0
	30	3	ICSI	17	0	0	16	3	3	3
3	29	6	IVF	19	2	3	12	2	2	0
	29	6	ICSI	18	0	2	16	0	0	0
	30	7	ICSI^a^	14	1	4	9	0	0	0

Abbreviations: *IVF*, in vitro fertilization; *ICSI*, intracytoplasmic sperm injection; *GV*, germinal vesicle; *MII*, metaphase II

^a^Half of MII were treated by assisted oocyte activation (AOA)

^b^The unfertilized MII were treated with remedial ICSI

All three patients were younger than 30 years and were infertile for longer than 3 years without an explicit etiology. They had normal menstruation, and their male partners had a normal sperm count, morphological features, and motility. The male partner of patient I had previously impregnated another woman. To further exclude male causes of infertility, donated IVM MII oocytes from normal patients were injected with sperm that were acquired from the other two men. These oocytes were fertilized, which excluded male causes of fertility.

Patient I had three failed artificial inseminations with her husband. This patient then underwent one IVF cycle, and 14 retrieved MII oocytes were not fertilized. In the next ICSI treatment, 11 MII oocytes were acquired, and half of the oocytes were artificially activated with assisted oocyte activation (AOA), but all of them failed to achieve PN formation. Patient II had two failed ICSI attempts, including one remedial ICSI attempt. In these attempts, 35 MII oocytes were retrieved, and 4 of them were fertilized, including three 2PN zygotes and one 3PN zygote. On day 3, the three 2PN zygotes developed into two four-cell embryos and one five-cell embryo. However, these embryos were all fragmented. The affected individual in family 3 underwent one IVF attempt and two ICSI attempts in which 37 MII oocytes were achieved. In the first attempt at ICSI, two 2PN fertilized oocytes fragmented shortly after PN formation, and the other oocytes failed to achieve PN formation. In the third attempt at ICSI, half of the MII oocytes were also treated with AOA, but they were not fertilized. In conclusion, patient I had TFF, but oocytes from patients II and III had low fertilization rates (3/35 and 2/37, respectively), but these early embryos failed to develop normally.

### Morphology and expression of the *WEE2* protein in human oocytes

**Fig. 2:**
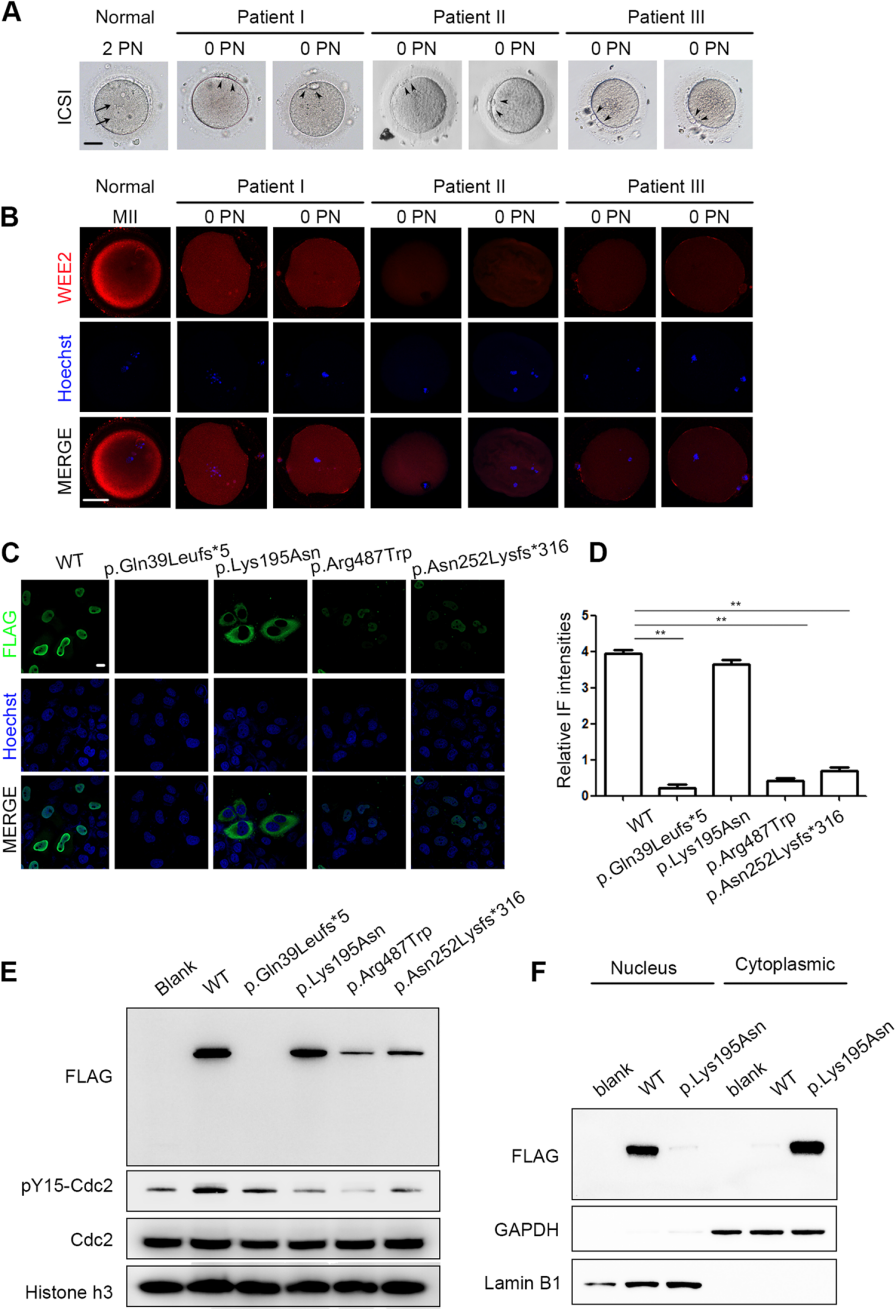
Characteristics and WEE2 levels of affected oocytes and mutations’ effects on subcellular localization and WEE2 expression levels in HeLa cells. (**A**) Light microscopic images of a normal zygote (left) and oocytes with failed fertilization at day 1 after ICSI. The black arrowheads represent polar bodies, and the black arrows represent pronuclei. Scale bar = 40 μm. (**B**) Immunostaining of WEE2 in normal oocytes from a donor and unfertilized oocytes from affected patients. Oocytes were immunolabeled with WEE2 antibody (red) and Hoechst (blue). Scale bar = 50 μm. MII, metaphase II; PN, pronucleus; ICSI, intracytoplasmic sperm injection. (**C**) Immunostaining of FLAG antibody (green) and Hoechst (blue) in HeLa cells transfected with wild-type or mutant plasmids. Scale bar = 10 μm. (**D**) Relative intensities of immunofluorescence signals in HeLa cells. **p < 0.01. (**E**) Western blotting shows the effects of wild-type and mutant constructs on WEE2 protein levels and tyrosine 15 phosphorylation of Cdc2 in HeLa cells. (**F**) Levels of Flag-WEE2 protein in the nuclear and cytoplasmic compartments after wild-type and mutant plasmid transfection in HeLa cells. Lamin B1 and GAPDH primary antibodies were used as internal controls for the nuclear and cytoplasmic compartments, respectively

The phenotypes of oocytes from the affected patients are shown in Fig. 2A. Oocytes with *WEE2* gene mutations had very little PN formation after insemination of sperm. The IF signal of the WEE2 protein in unfertilized oocytes (0PN) from patient I was still detectable, and the signals of oocytes from the other two patients were much weaker than those of donated IVM oocytes from healthy women (Fig. 2B).

### Expression and localization of the mutant *WEE2* protein in HeLa cells

Among the six variants, the p.Glu75Valfs*6 and p.His337Tyrfs*24 variants have been proven to dramatically reduce WEE2 protein levels [7]. Thus, we conducted in vitro experiments on only the p.Lys195Asn, p.Gln39Leufs*5, p.Arg487Trp, and p.Asn252Lysfs*316 mutations. The p.Gln39Leufs*5 mutation caused a dramatic loss of WEE2 protein expression. Furthermore, the p.Arg487Trp and p.Asn252Lysfs*316 mutations caused reduced expression of the WEE2 protein, as shown by immunofluorescence (Fig. 2C and D) and western blotting (Fig. 2E). Interestingly, the p.Lys195Asn mutation induced more nuclear export than the wild-type WEE2 protein, which was mostly in the nucleus. Similar to the immunofluorescence results, the compartment fractionation experiment showed that the majority of the p.Lys195Asn protein was detected in the cytoplasm instead of the nucleus (Fig. 2F). The nuclear localization of the other three mutants was not changed.

### Reduced inhibitory effects of the mutant *WEE2* protein on the cell cycle

**Fig. 3:**
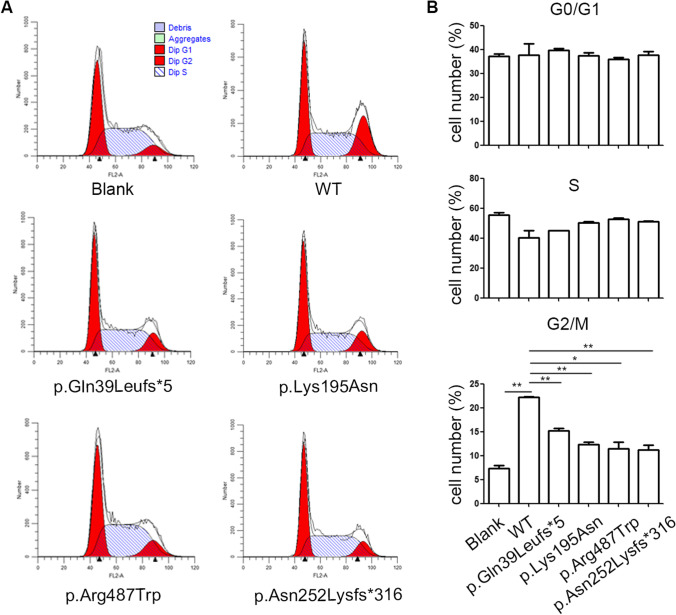
Inhibitory effects of *WEE2* mutation variants on the cell cycle. (**A**) Overexpression of wild-type and mutant *WEE2* variants induced G2/M arrest of the cell cycle in HeLa cells. (**B**) Summary of different phases of the cell cycle by flow cytometric analysis. *p < 0.05, **p < 0.01

Flow cytometry was used to evaluate the inhibitory effects of overexpression of the wild-type and mutant WEE2 protein on the cell cycle in HeLa cells (Fig. 3). In HeLa cells transfected with wild-type WEE2 plasmids, the ratio of cells that were arrested in the G2/M phase was significantly higher than that in the nontransfected (blank) group (*p* < 0.01). However, the ratio of G2/M arrested cells in the p.Gln39Leufs*5, p.Arg487Trp, p.Asn252Lysfs*316, and p.Lys195Asn groups was slightly higher than that in the blank group and significantly lower than that in the wild-type group. The ratio of cells that were arrested in the S and G0/G1 phases was not significantly different among the six groups.

## Discussion

In the present study, we found six *WEE2* gene mutations in three patients who suffered from primary infertility for longer than 2 years and experienced repeated ART failure because of fertilization failure.

The affected woman in family I had p.Glu75Valfs*6 and p.Lys195Asn compound heterozygous mutations. The p.Glu75Valfs*6 mutation site induced dramatic loss of WEE2 protein expression in affected oocytes [7]. The homozygous p.Lys195Asn mutation was also found in a similar patient whose oocytes expressed normal levels of the WEE2 protein, but the phosphorylation level of the Cdc2 protein was significantly reduced for unknown reasons [10]. Our study suggests that the p.Lys195Asn mutation site causes WEE2 protein translocation instead of reducing the protein amount in HeLa cells. Therefore, the WEE2 protein is unable to phosphorylate the Cdc2 protein in the nucleus, which induces a decrease in phosphorylation of the Cdc2 protein. Although the cellular structure of MII oocytes is different from that of HeLa cells, studies in mouse oocytes observed translocation of the WEE2 protein [9]. The mWee2 protein mostly stays in the nucleus to maintain meiotic arrest at the GV stage, and re-entry into meiosis requires mWee2 translocation from the nucleus to the cytoplasm, which also activates MPF [9]. If translocation of mWee2 occurs earlier in the GV stage, it may lead to leakage of arrest of GVs and re-entry into meiosis I, inducing decreased oocyte fertility. In our routine IVF protocols, MII oocytes were acquired after 34–36 h of human chorionic gonadotrophin (hCG) injection. Based on published studies and our present study, we are unsure whether the process of GV arrest or meiosis re-entry is normal in patients with pathogenic *WEE2* mutations. We assumed that disturbed subcellular localization of WEE2 also interfered with the fertilization process.

The patients in families 2 and 3 had slightly different situations, with 2PN formation, although the rate was extremely low (8.6% and 5.4%, respectively). Although normal embryos appeared to be acquired, they fragmented soon after observation of a PN. In published articles, four patients with WEE2 mutations [10, 12, 14, 16] also had a low rate of 2PN formation. All of these 2PN failed to develop normal embryos. In conclusion, we consider the WEE2 protein to play roles not only in fertilization but also in early embryo development. We found that the mutant plasmids (p.Arg487Trp from patient II and p.Asn252Lysfs*316 from patient III) endowed the oocytes with residual protein levels, which may confer partial fertilization ability. However, the extrinsic mechanism by which WEE2 and phosphorylated Cdc2 affect early embryo development still needs further clinical and experimental investigations.

Other gene mutations were also reported to be associated with failure of fertilization, as summarized by a recent review [17]. However, those genes are usually associated with different clinical symptoms. For example, mutations in *TLE6* (MIM: 612,399) are associated with failure of fertilization but are also related to the earliest embryonic cleavage [18]. *TUBB8* (MIM: 616,768) and *PATL2* (MIM: 614,661) are associated with various phenotypes, including meiotic arrest, dysfunction of fertilization, and developmental disorder of blastocytes [19, 20]. Additionally, *ZP1* (MIM: 195,000) and *ZP2* (MIM: 182,888) gene mutations are associated with abnormalities of the ZP, which induces fertilization failure [21]. Different genes have specific or varying effects on human reproductive processes. Depending on the clinical symptoms, researchers should choose specific gene sequencing or whole-exome sequencing to discover genetic causes of infertility that may lead to precise diagnoses and treatments.

In conclusion, we report 6 compound heterozygous mutation variants in the *WEE2* gene that might be related to failure of fertilization. These mutations contribute to functional loss of the WEE2 protein, as shown by in vitro experiments. Our study provides new evidence that the *WEE2* gene has a high mutation rate in patients with TFF and low fertilization and plays a major role in human fertilization.

## Supplementary Information

Below is the link to the electronic supplementary material.

Supplementary file1 (DOCX 24 KB)
