# Supplementary material for: Novel WEE2 compound heterozygous mutations identified in patients with fertilization failure or poor fertilization
Source: J Assist Reprod Genet. 2021 Sep 3;38(11):2861–9. doi: 10.1007/s10815-021-02285-z (PMC8608989; doi:10.1007/s10815-021-02285-z)
Supplement: Supplementary file 1 — Supplementary file1 (DOCX 24 KB) [file 10815_2021_2285_MOESM1_ESM.docx]

**Table S1. Whole exome sequencing data of 3 patients with WEE2 mutation.**

| **Sample ID** | **Patient 1** | **Patient 2** | **Patient 3** |
| --- | --- | --- | --- |
| Accurate mapped bases (Mb) | 12094.94 | 11904.81 | 18604.39 |
| Accurate mapping rate (%) | 98.6 | 97.31 | 99.63 |
| Target size | 58231156 | 58231156 | 62984579 |
| Target covered size | 58066187 | 58074074 | 62788185 |
| Coverage rate (%) | 99.72 | 99.73 | 99.69 |
| Target mean depth | 140.25 | 143.55 | 144.01 |
| 4X coverage rate (%) | 99.6 | 99.61 | 99.57 |
| 10X coverage rate (%) | 99.21 | 99.26 | 99.31 |
| 20X coverage rate (%) | 98.12 | 98.29 | 98.59 |

**Table S2. Primers of WEE2 mutation sites for Sanger analysis.**

| **Family** | **Mutation variant** | **Direction** | **Sequences (5’-3’)** | **Target size (bp)** |
| --- | --- | --- | --- | --- |
| I | c.220_223delAAAG | Forward | CCTCCCTGCTTCTGTAGGTTC | 637 |
|  |  | Reverse | CTGGCACCAGTGGTTTTGAGA |  |
|  | c.G585C | Forward | TCGTTCCCAGAATCTTGGCT | 669 |
|  |  | Reverse | CTAGCTGAGAACACAGACCCTG |  |
| II | c.115_116insT | Forward | CCTCCCTGCTTCTGTAGGTTC | 637 |
|  |  | Reverse | CTGGCACCAGTGGTTTTGAGA |  |
|  | c.C1459T | Forward | TGGTGCTACCAGACATGGAC | 467 |
|  |  | Reverse | GTGGGCAGGGACCTATGTATT |  |
| III | c.756_758delTGA | Forward | CCAACATGGCTTCCCGCTA | 254 |
|  |  | Reverse | TGATTTTTAATGACATCGGTGCTT |  |
|  | c.1006_1007insTA | Forward | ACTCATACTGTGGGGCTGTTC | 212 |
|  |  | Reverse | AAGTGGCAGAAGGGAATCACA |  |

**Table S3. Primers of WEE2 mutation sites for plasmid mutagenesis.**

| **Mutation site** | **Direction** | **Sequences (5’-3’)** |
| --- | --- | --- |
| c.G585C | Forward | cgtaaaacacatctgttggcaggcagccctcc |
|  | Reverse | ggagggctgcctgccaacagatgtgttttacg |
| c.115_116insT | Forward | ggaggcttcgagcctaaaccccagagaag |
|  | Reverse | cttctctggggtttaggctcgaagcctcc |
| c.C1459T | Forward | tttcccagggaaggccagagaactgtatttctggc |
|  | Reverse | gccagaaatacagttctctggccttccctgggaaa |
| c.756_758delTGA | Forward | ctatgaaaacttttacagaattatcaaagaattcggctttgcatgaagttta |
|  | Reverse | taaacttcatgcaaagccgaattctttgataattctgtaaaagttttcatag |
